# Choix thérapeutiques des hypertendus et diabétiques en milieu rural : Une étude mixte dans deux zones de santé de l’Est de la République Démocratique du Congo

**DOI:** 10.4102/phcfm.v14i1.3004

**Published:** 2022-09-29

**Authors:** Aimé C. Mwana-wabene, Samuel M. Lwamushi, Christian M. Eboma, Pacifique M-B. Lyab, Bonfils Cheruga, Hermès Karemere, Albert T. Mwembo, Ghislain B. Balaluka, Faustin C. Mukalenge

**Affiliations:** 1 École Régionale de Santé Publique, Faculté de Médecine, Université Catholique de Bukavu, Bukavu, The Democratic Republic of the Congo; 2 École Régionale de Santé Publique, Faculté de Médecine, Université de Lubumbashi, Lubumbashi, The Democratic Republic of the Congo; 3 Renforcement Institutionnel des politiques de Santé basées sur les évidences en R. D. Congo, Lubumbashi, The Democratic Republic of the Congo; 4 Renforcement institutionnel des politiques de santé basées sur les évidences en R. D. Congo, Bukavu, The Democratic Republic of the Congo; 5 Centre de Connaissances en Santé au Congo, Kinshasa, The Democratic Republic of the Congo; 6 Ecole de Santé Publique, Faculté de Médecine, Université de Lubumbashi, Lubumbashi, The Democratic Republic of the Congo

**Keywords:** Therapeutic choices, diabetics, hypertensives, rural areas, South Kivu

## Abstract

**Therapeutic choices of hypertensive and diabetics in rural areas: A mixed study in two health zones in the East of the Democratic Republic of Congo**.

**Background:**

One third of patients in the Democratic Republic of Congo (DRC) do not use the formal health system to access healthcare.

**Aim:**

In this manuscript we analyse the therapeutic decisions of hypertensive and diabetic patients in rural eastern DRC and the reasons for these decisions.

**Setting:**

The study was conduct in two health zones (HZ) in South Kivu (Bagira and Walungu), DRC.

**Methods:**

A mixed-methods convergent study was conducted from November 2018 to December 2018. Quantitative data were collected using a questionnaire and qualitative data were collected using focus groups. The quantitative data were analysed using descriptive statistics and a Fischer exact test, while the qualitative data were analysed using thematic analysis.

**Results:**

Out of 382 subjects declaring a chronic pathology, hypertensives and diabetics represented 21.5% and 7.9%, respectively. Health facilities were the first therapeutic choice of the chronically affected persons. The alternative therapeutic choices found were the use of prayer rooms, consultation with traditional healers and self-medication. Poverty, ignorance, the pharmaceutical business, and the socio-cultural dimension of the disease are the main causes of alternative therapeutic choices for hypertensives and diabetics.

**Conclusion:**

To ensure appropriate care for patients with chronic diseases in rural areas, it is important to establish a bridge of regulated collaboration between the formal and informal health sector.

## Introduction

Les maladies non transmissibles (MNT) constituent un problème majeur de santé publique dans le monde de par la charge socio-économique qu’elles engendrent^1^. Chaque année, plus de 40 millions de personnes meurent dans le monde et 70% de ces décès sont attribués aux MNT^2,3^. On estime qu’environ 80% de décès liés aux MNT sont enregistrés dans des pays à bas et moyens revenus (PBMR)^2,3,4,5^.

Le traitement des cinq MNT les plus fréquentes, à savoir, les maladies cardiovasculaires, le diabète, le cancer, les affections des voies respiratoires et les troubles musculo-squelettiques occasionne environ 40% des coûts directs de la santé dans le monde^1^. En plus de ces coûts directs de traitement, la société supporte également des coûts indirects substantiels. Il s’agit principalement de pertes de productivité résultant de la maladie ou d’un décès prématuré^6^. Ce fardeau socio-professionnel et économique est aussi représenté par la baisse de performance au travail et les absences prolongées s’accumulent dans le secteur professionnel, le temps des proches et des amis qui soignent gratuitement une personne malade^1^.

L’Afrique subsaharienne, à l’instar de la plupart des PBMR, traverse une transition épidémiologique rapide au cours de laquelle les MNT commencent à prendre de l’ampleur^7,8^. Une récente revue de la littérature réalisée sur les MNT dans les plusieurs pays en Afrique subsaharienne a montré des prévalences respectivement de 0.0% à 16.0%, 6.0% à 48.0% et 0.4% à 43.0% pour le diabète sucré, l’hypertension artérielle et l’obésité^8^. Cette augmentation des MNT est due en grande partie à la transition nutritionnelle avec la modification de facteurs environnementaux ayant un impact sur la nutrition (baisse de l’activité physique et un changement des comportements alimentaires, avec une consommation plus importante de produits industriels au détriment de l’alimentation traditionnelle)^9,10,11,12^.

A côté de l’augmentation de la prévalence dans les PBMR, s’ajoute les conflits armés entrainant une crise chronique qui altère la santé de la population générale^13^. Cette crise entraine également un stress chronique pouvant provoquer la survenue des MNT et être un obstacle aux stratégies de lutte contre les MNT tel que l’a montré une revue systématique en 2019^14^. Aussi, une étude menée en République Centre Africaine sur les déplacés internes, fuyant les zones de conflits armés, a trouvé une prévalence de 34.40% pour les MNT avec en tête l’hypertension (18.99%), suivie du diabète sucré (15.08%)^15^.

En République Démocratique du Congo (RDC), l’Organisation Mondiale de la santé (OMS) a estimé que les maladies non transmissibles étaient à l’origine de 23% de tous les décès en 2014^16^. Le système de santé de la RDC prévoit trois échelons dont deux au niveau opérationnel (un réseau des centres de santé (CS) avec un hôpital général de référence (HGR) qui fonctionnent dans la zone de santé) et un troisième échelon couvert par un hôpital universitaire et/ou provincial.

Compte tenu de la grandeur du pays, le système a intégré des structures de compromis au niveau opérationnel (Poste de santé : PS et Centre de santé de Référence : CSR) dont les paquets d’activités ne sont pas standardisés^17^. On estime à plus de 5 millions le nombre de décès associés aux crises humanitaires dans l’Est de la RDC^18,19,20^. C’est une région instable avec des mouvements importants des déplacés fuyant les zones de conflits. En fin 2017, le Sud-Kivu a enregistré 647 000 personnes déplacées, faisant d’elle l’une des provinces les plus touchées par le mouvement des populations en RDC^21^. Par ailleurs, la prévalence des maladies chroniques, notamment l’hypertension et le diabète sucré présentent une tendance à l’augmentation rapide aussi bien en milieu rural qu’en milieu urbain. D’après des études récentes menées dans la région, les prévalences de l’hypertension artérielle (HTA) et du diabète sucré étaient respectivement de 19.0% et de 3.5% dans la population générale^22,23^. Depuis 2015, le gouvernement congolais, en collaboration avec plusieurs partenaires bilatéraux et multilatéraux de développement, ont contribué à la mise en place à travers un plan national de développement sanitaire réunissant des stratégies pour réduire d’un tiers les décès prématurés dus aux MNT au cours des quinze prochaines années comme le prévoient les Objectifs de Développement Durable (ODD)^24^. Pour y arriver, il est capital de connaitre la proportion de ces différentes maladies chroniques non transmissibles dans la communauté ainsi que les différents itinéraires thérapeutiques auxquels recourent ces patients tout au long de leur maladie. Une récente étude transversale menée au sein de la population générale en milieu rural au Sud-Kivu a trouvé que 5.3% et 7.9% des patients utilisent respectivement les chambres de prière et l’automédication comme premier moyen de recourir aux soins^25^ lorsqu’ils sont malades. Mais, aucune étude au Sud-Kivu n’a encore mis l’accent sur les choix thérapeutiques des malades avec MNT au Sud-Kivu alors qu’on remarque que le taux d’utilisation des services curatifs reste faible ces cinq dernières années (inférieur à 0.5 NC/hab./an)^26^.

La problématique du choix thérapeutique des patients avec MNT en milieu rural dans ce contexte reste alors une question pertinente vu la présence de la médecine parallèle, mieux accessible que la médecine moderne dans ce milieu^27^ et le fardeau socio-économique et professionnel que ces maladies entrainent.

Notre étude vise à identifier les choix thérapeutiques des diabétiques et hypertendus en milieu rural au Sud-Kivu et à dégager les motivations justifiant ce choix.

## Méthodologie

### Présentation du milieu et période de l’étude

L’Est de la RDC, plus précisément les hautes terres de la crête Congo-Nil constituant la partie orientale des deux provinces du Nord-Kivu (capitale Goma) et du Sud-Kivu (capitale Bukavu), est, depuis la guerre du Rwanda en 1994, l’épicentre des conflits persistants, avec leur cortège de souffrances, leurs victimes civiles, leurs cohortes de réfugiés, sa confusion militaire, leurs crises humanitaires à répétition qui ont des conséquences sur la santé des populations^18,19,20^. Les différentes enquêtes de la santé confirment cet état de vulnérabilité dans lequel vit la population congolaise^28^.

L’étude a été menée durant deux mois (de novembre 2018 à décembre 2018) dans la province du Sud-Kivu à l’Est de la RDC, dans deux zones de santé (ZS) : la ZS rurale de Walungu et la ZS urbaine de Bagira. Ces deux ZS ont été sélectionnées par convenance du fait de leur implication dans la mise en œuvre d’un projet interuniversitaire de recherche-action orienté vers le développement des soins centrés sur la personne. La ZS de Walungu couvrait une population de 252 269 habitants avec 23 aires de santé, et celle de Bagira une population de 139 361 habitants en 2018 et comptait 8 aires de santé^29^.

### Type d’étude

Il s’agit d’une étude transversale dans laquelle nous avons utilisé une approche mixte type convergente^30^ : la partie qualitative est transversale et la qualitative est exploratoire descriptive. Les données qualitatives et quantitatives ont été récoltées concomitamment, analysées de façon séparée et enfin, une triangulation de ces données a été réalisée. Ce design nous a permis d’obtenir une vue complète sur le choix thérapeutique des hypertendus et diabétiques à partir de deux bases de données afin de corroborer les résultats issus de ces deux différentes méthodes^30,31^.

### Étude quantitative

#### Échantillonnage et population d’étude

- **Sélection des aires de santé**

Nous avons exploité des rapports annuels des ZS pour identifier les aires de santé ayant rapporté les cas d’hypertension et de diabète, soit 8 aires de santé (desservant une population de 127 816 habitants) à Walungu et 4 (desservant une population de 82 284 habitants) à Bagira.

- **Calcul de la taille d’échantillon**

Nous avons d’abord calculé la taille minimale d’échantillon grâce à la formule classique de Schwartz ci-après :


$$n=\frac{p(1-p)z^{2}}{d^{2}}$$ [Eqn 1]

*p* = prévalence du diabète sucré ou de l’hypertension artérielle au Sud-Kivu. Nous avons considéré la fréquence de l’HTA dans une étude populationnelle (étude de cohorte d’une population de 7157 sujets) au Sud-Kivu qui était de 12.1%^32^ en 2015, comparativement à la prévalence de 41.0% dans la même population en 2011^33^:


$$\begin{array}{l} z=\text{valeur type du niveau de cinfiance de }95.0\%,\text{ estimé à }1.96\%\\ d=\text{marge d’erreur de }5.0\% \end{array}$$ [Eqn 2]

La taille minimale d’échantillon calculée était de 172 sujets. Cette taille d’échantillon a été corrigée par l’effet grappe, en la multipliant par 2 et en une valeur de prévision de non réponse de 11% ; ce qui correspond à une taille d’échantillon de 382 sujets.

- **Sélection des ménages**

Les ménages ont été identifiés par village en milieu rural (Walungu) ou par avenue en milieu urbain (Bagira). Pour chaque aire de santé, le premier village (ou avenue) a été choisi de manière aléatoire sur la liste des villages ou avenues établie par chaque CS. Une fois dans les villages et avenues, l’équipe d’enquêteurs s’est placée au milieu du village ou de l’avenue avant de tirer aléatoirement une direction à suivre grâce à la pointe du stylo jeté par terre. Après avoir compté le nombre de ménages dans la direction choisie, le premier ménage à enquêter était désigné de manière aléatoire sur la direction à partir des numéros de série des billets de banque en possession par un des membres de l’équipe d’enquêteurs.

Dans un ménage, les patients souffrant d’une maladie depuis au moins 6 mois et âgés de plus de 18 ans ont été inclus dans l’étude, sur base d’une preuve consistant à la notification dans leur carnet médical pour ceux qui fréquentent les structures de soins et/ou à une déclaration verbale de l’accompagnant pour ceux qui fréquentent les tradipraticiens. Un ménage visité qui n’avait pas de sujet remplissant ce critère a été remplacé par le ménage le plus proche. Et le recrutement s’est poursuivi dans les villages ou avenues voisins jusqu’à atteindre le quota établi pour chaque aire de santé.

#### Collecte des données

La collecte des données a été assurée par cinq médecins préalablement formés. Un prétest pour la compréhension du questionnaire a été réalisé dans une autre ZS non concernée par cette étude.

Nous avons collecté les données sociodémographiques : âge, genre, état civil, niveau d’études, et profession. Pour valider le type de pathologie chronique, nous nous sommes servis du carnet médical du patient : L’HTA était confirmée si au moins une mesure anormale de la TA était signalée (TAS ≥ 140 et/ou TAD ≥ 90 mm Hg)^34^ et le diabète sucré était validé si au moins une valeur anormale de la glycémie était notifiée (Glycémie à jeun ≥ 126 mg/dL sur la prise de glycémie capillaire)^35^ dans le carnet médical des patients. Les autres pathologies chroniques n’ont pas été classées parce qu’il n’existait pas de preuve objective pour le diagnostic. Parmi ces pathologies nous avons considéré : l’asthme bronchique, l’épilepsie, la gastrite chronique, la cirrhose hépatique, l’insuffisance cardiaque chronique, le rhumatisme articulaire ainsi que la goutte.

Concernant le recours aux soins, nous avons recherché et enregistré la dernière structure de soins fréquentée (y compris la médecine traditionnelle et les chambres de prière) par le sujet au cours des six derniers mois.

L’automédication a été retenue chaque fois qu’un sujet déclarait avoir consommé des médicaments de lui-même, obtenus directement de la maison à partir de la pharmacie familiale, des officines pharmaceutiques ou du marché chez des vendeurs ambulants de médicaments. Le recours aux familiers ou aux amis professionnels de santé en privé ou à leurs domiciles a été considéré comme un mode de recours thérapeutique à part^36^.

#### Analyse des données

Les données recueillies ont été saisies sur une feuille Excel 2016. L’analyse a été faite dans le logiciel Stata version 14. Les variables qualitatives ont été résumées par la fréquence et la proportion tandis que les variables quantitatives ont été résumées par la moyenne et l’écart-type. Le test X^2^ de Pearson ou celui de Ficher Exact a été effectué pour comparer les proportions de différentes variables catégorielles en fonction des zones de santé. Un *p*-valeur de < 0.05 a été considéré comme statistiquement significatif.

### Étude qualitative

#### Échantillonnage

Nous avons appliqué un échantillonnage raisonné ciblant différents acteurs du système de santé (formels et informels) : prestataires de soins, présidents des comités de santé, tradipraticiens, responsables des chambres de prière, responsables des officines pharmaceutiques et les malades.

La sélection des responsables d’officines, les tradipraticiens et les responsables de chambres de prière a été faite par la méthode de boule de neige^37^.

#### Collecte des données

Les données ont été collectées grâce à des focus groups de ces différents groupes des personnes sélectionnées. Les focus groups comprenaient 8 à 10 personnes ayant les mêmes qualifications tel que décrit dans le tableau au-dessus (Tableau 1).

**TABLEAU 1:** Caractéristiques des interviewés.

Acteurs du système de santé	Nombre
Prestataires de soins	10
Présidents des comités de santé	8
Tradipraticiens	9
Responsables de chambre de prière	8
Responsables des officines pharmaceutiques	8
Malades	10

Deux médecins formés dans la collecte des données qualitatives, dont une personne modérant le débat et l’autre prenant les notes et vérifiant l’enregistreur, ont conduit ces focus groups. L’entretien était enregistré avec l’accord préalable des participants. Les fichiers audio issus de ces entretiens ont ensuite été transcrits par les deux médecins. Ces focus groups avaient une durée moyenne de 50 minutes.

Le guide d’entretien, rédigé initialement en français a été traduit en langues locales (swahili et mashi pour les deux ZS). Le processus de traduction a été fait en deux étapes : la première consistait en une traduction en langues locales et la deuxième consistait à retraduire le questionnaire en français par l’École de langue de l’université Catholique de Bukavu (UCB). Les trois versions du questionnaire ont été utilisées durant l’enquête selon la préférence linguistique du répondant (français, mashi et swahili).

Les questions abordées dans ce guide étaient focalisées sur les raisons et les motivations des choix thérapeutiques des patients (automédication, utilisation des chambres de prière ou des tradipraticiens).

#### Analyse des données

Les données qualitatives provenant des enregistrements audio ont été transcrites mot à mot. Quatre transcripts supposés être les plus informatifs ont été analysés manuellement puis les autres ont été analysés grâce au logiciel Nvivo 15 pour synthétiser les opinions des interrogés sur la motivation du choix des différents itinéraires thérapeutiques des patients.

Les propos des répondants choisis dans la présentation des résultats sont présentés en italique et entre guillemets. Pour nommer les propos, l’initial du groupe cible, le numéro correspondant à son entretien : les patients sont nommés P1 jusqu’à P16, les prestataires des soins Pr1 jusqu’à Pr10, les tradipraticien T1 jusqu’à T10, les responsables des officines pharmaceutiques (RespFP1 jusqu’à RespFP10) ; et la catégorie d’appartenance au système de santé (f pour informel et i pour informel) ont été choisi, en s’inspirant du critère de COREQ^38^.

### Considérations éthiques

Le protocole de l’étude a été approuvé par le Comité d’Éthique de l’Université Catholique de Bukavu. L’autorisation écrite pour la collecte des données avait été délivrée préalablement par le médecin chef de Division Provinciale de la Santé du Sud Kivu. La participation des différentes personnes interrogées a été conditionnée par leur consentement verbal éclairé et libre avec une garantie du respect de l’anonymat et de la confidentialité.

## Résultats

### Caractéristiques socio-économiques des patients au sein des ménages

La moyenne d’âge (DS) des patients enquêtés était de 56 ± 2 ans et de 54 ± 1.9 ans respectivement à Bagira et à Walungu avec un genre ratio M/F d’environ 1.71 pour les deux ZS. Dans les deux ZS, environ la moitié des patients n’avaient pas de travail et étaient non scolarisés. Seul 3% des patients avaient atteint un niveau d’éducation universitaire dans les 2 zones alors que la proportion des mariés était à 61.0% (Tableau 2).

**TABLEAU 2:** Caractéristiques socio-économiques des malades enquêtés.

Variables	Zone de sante								Total			
	Bagira				Walungu				Moyen	±écart-type	*n*	%
	Moyen	±écart-type	*n*	%	Moyen	±écart-type	*n*	%				
**Âge (*n* = 382)**	56	±2 ans	-	-	54	±1.9	-	-	55	±1.9	-	-
**Genre (*n* = 382)**												
Féminin	-	-	62	33.7	-	-	79	39.9	-	-	141	36.9
**Statut matrimonial (*n* = 382)**												
Célibataire/séparé	-	-	78	42.4	-	-	71	35.9	-	-	149	39.0
Marié(e)s	-	-	106	57.6	-	-	127	64.1	-	-	233	61.0
**Profession (*n* = 382)**					-	-			-	-		
Sans emploi	-	-	105	57.0	-	-	80	40.4	-	-	185	48.5
Indépendant	-	-	63	34.2	-	-	105	53.0	-	-	168	43.9
Salarié	-	-	16	8.8	-	-	13	6.6	-	-	29	7.6
**Niveau d’instruction (*n* = 382)**												
Non scolarisé	-	-	71	38.6	-	-	102	51.5	-	-	173	45.3
Primaire	-	-	48	26.1	-	-	52	26.2	-	-	100	26.1
Secondaire	-	-	55	29.9	-	-	42	21.2	-	-	97	25.4
Universitaire	-	-	10	5.4	-	-	2	1.0	-	-	12	3.1

### Fréquence de l’hypertension et du diabète

Sur 382 personnes enquêtées ayant rapporté avoir une pathologie chronique, 82 (soit 21.5%) ont de l’hypertension artérielle et 30 (soit 79) ont le diabète sucré (Tableau 3).

**TABLEAU 3:** Fréquence de l’hypertension et du diabète dans les zone de santé de Bagira et Walungu (*n* = 382).

Pathologies chroniques	Global	Bagira		Walungu	
		*n*	%	*n*	%
HTA	82	45	54.9	37	45.1
Diabète sucré	30	23	76.7	7	23.3
Autres	270	116	43.0	154	57.0

HTS, de l’hypertension artérielle.

Ces deux pathologies sont fréquemment trouvées dans la zone de santé de Bagira tandis que les autres pathologies chroniques ont été répertoriées dans la zone de santé de Walungu.

### Choix thérapeutiques des diabétiques et hypertendus dans les zones de santé

En termes de choix thérapeutique, le Tableau 4 indique que la proportion des patients ayant des pathologies chroniques recourent aux FOSA intégrées comme choix thérapeutique. Les malades avec pathologies chroniques, y compris les diabétiques et les hypertendus, consultent en majorité les formations sanitaires intégrées dans le système de santé. En dehors des formations sanitaires, ces patients utilisent comme autre itinéraire thérapeutique les chambres de prière et les tradipraticiens. Les diabétiques et les hypertendus par contre consultent moins ces deux itinéraires thérapeutiques, avec simultanément des proportions de < 10% et < 20%.

**TABLEAU 4:** Choix thérapeutiques des patients dans les deux zones de santé.

Choix thérapeutiques	HTA				*p*	Diabète				*p*
	Oui		Non			Oui		Non		
	*n*	%	*n*	%		*n*	%	*n*	%	
**FOSA intégrées (*n* = 382)**										
Oui	57	21.9	203	78.1	0.75	21	8.1	239	91.9	0.81
Non	25	20.5	97	79.5		9	7.4	113	92.6	
**FOSA Privées (*n* = 382)**										
Oui	18	31.0	40	69.0	0.054	3	5.2	55	94.8	0.41
Non	64	19.8	260	80.2		27	8.3	297	91.7	
**Tradipraticiens (*n* = 382)**										
Oui	14	19.2	59	80.8	0.60	6	8.2	67	91.8	0.34
Non	68	22.0	241	78.0		24	7.8	285	92.2	
**Chambres de prière (*n* = 382)**										
Oui	14	13.3	91	86.7	**0.017**	6	8.2	99	94.3	0.34
Non	68	24.5	209	75.5		24	8.7	253	91.3	

### Perceptions des interrogés concernant la motivation du choix thérapeutique des patients

Les acteurs œuvrant dans ces chambres de prière exploitent la dimension socio-culturelle de la maladie pour attirer les personnes malades à les consulter à des fins lucratives. Ces agents peuvent être des pasteurs ou alors des membres de la communauté :

‘… Dans ce milieu nous avons d’autres pasteurs qui trompent des gens et demandent de l’argent avant la prière, voilà pourquoi on les a invités ils ne sont pas venus …’ (Past2i)

‘ … Dans mon aire de santé il y a une maman qui a transformé sa maison en une chambre de prière et elle reçoit les gens venant de tous les coins du Sud-Kivu …’ (Pr7f)

L’autre raison faisant que les chambres de prière prennent de l’ampleur en termes de choix thérapeutique de première intention est le fait que l’autorité sanitaire censée réguler le secteur ne fait pas son travail :

‘… Nous on pense qu’avec les chambres de prière qui ont des lits même pour des malades, la santé des gens est en insécurité, malheureusement nous n’avons pas un gouvernement responsable pour poursuivre ces gens … il y en a même qui ont des tarifs.’ (Pr3f)

A part les chambres de prières, les tradipraticiens absorbent une partie non négligeable des malades chroniques. Ces derniers exploitent non seulement la dimension « mystique » de la maladie mais aussi se servent de l’ignorance et de la pauvreté des malades pour vendre leurs services :

‘On soigne la tension artérielle ; on donne au malade des médicaments à prendre au repas. On lui demande d’acheter des grains de courge et des bananes de sorte que de tension soit éliminée … C’est ça notre chimie.’ (Tr8i)

‘… on donne les médicaments et un bon suivi dure 2 mois ou 120 jours tout au plus si le patient suit les conseils donnés. Ça dépend aussi du type de diabète. Si c’est lié au surpoids, il faut aussi lutter contre l’obésité au même moment où tu modifies le traitement. Si la personne a déjà pris de l’insuline, il faut vider toutes ces insulines de son corps … Tu ne peux pas utiliser le jus de sucre comme véhicule pour eux. Il faut donner un produit qui est sous forme de poudre et la condition est de le prendre à l’état tiède sans mettre du sucre …’ (Tr4i)

Un autre choix thérapeutique ayant surgi dans les discussions de groupe était l’automédication. Parmi les raisons ayant poussé les patients à utiliser l’automédication figuraient.

Premièrement l’influence de la pauvreté et de la proximité avec des professionnels de santé qui conseillent des médicaments aux patients :

‘… Quand je me sens malade, ça ne sert à rien d’aller faire une consultation car elle va me demander assez d’argent pour une prescription finale de paracétamol que je peux avoir chez moi ou acheter au marché …’ (P5f)

‘… Non à côté de chez moi nous avons un médecin mais qui n’a pas de travail, et il est bien parfois quand je sens des malaises au-delà de mon diabète, je vais auprès de lui ; il me prescrit les médicaments et je vais acheter à la pharmacie …’ (P8f)

Deuxièmement, le fait que des responsables d’officines publiques visaient à écouler leurs médicaments, considérés comme marchandises, a déclaré l’informateur:

‘… Nous avons nos officines pharmaceutiques qui constituent notre activité génératrice des revenus … on ne peut pas refuser de l’argent d’un client …’ (RespFP3i)

Par contre, le fait de prendre conscience de sa maladie et du fait qu’elle nécessite un traitement continu, pousse certains diabétiques à renouveler leur stock de médicament sans prescription médicale dans les officines :

‘… Je connais déjà ma maladie … je souffre du diabète, et pour me procurer de l’insuline je n’ai plus besoin d’aller consulter c’est juste aller à la pharmacie pour acheter et je continue mon traitement …’ (P9f)

## Discussion

Cette étude avait pour objectif de déterminer la proportion des patients diabétiques et hypertendus chez les enquêtés vivant dans un milieu urbano-rural congolais, d’identifier leurs choix thérapeutiques et d’analyser les motivations justifiant ces choix.

### Les limites de l’étude

Notre étude a sélectionné les sujets reconnus déjà malades depuis plus de six mois. Ceci pourrait entraîner une surestimation de la proportion des malades diabétiques ou hypertendus dans ces deux ZS. Cependant, ceci a permis de sélectionner des anciens malades capables de renseigner sur les choix thérapeutiques et les motivations justifiant ces derniers.

Aussi, vu les limites de temps et de moyens, nous avions préférés faire des focus groups au lieu des entretiens individuels mais tout en prenant les groupes homogènes d’individus.

### Fréquence du diabète et de l’HTA

Dans notre étude, les hypertendus et les diabétiques représentent respectivement 21.5% et 7.9% de l’ensemble des personnes enquêtées dans les deux ZS. Concernant l’hypertension, ce résultat se rapproche de ceux trouvés au Burkina Faso, 17.3%^39^. Mais ils diffèrent de ceux trouvés en Guinée, Fouta-Djalon, 31.4%^40^ au Mali, 12.7%^41^ au Bénin^42^ et au Gabon, 38.5%^43^. Ces divergences peuvent être liées aux différentes approches méthodologiques (la population cible de l’étude).

Cependant, en comparant le milieu urbain (ZS de Bagira) avec le milieu rural (ZS de Walungu) dans notre étude, le diabète est significativement plus fréquent en milieu urbain (12.5%) qu’en milieu rural (3.5%). Nos résultats corroborent ceux d’autres auteurs africains qui ont montré qu’il y avait une disparité entre le milieu urbain et rural en termes de fardeau lié aux MNT avec une prédominance urbaine^32,39,44^. Ceci s’expliquerait par le fait qu’en milieu urbain, le style de vie comporte de plus en plus de facteurs de risque pour ces deux maladies, à savoir l’insuffisance d’activité physique, l’abus d’alcool et de cigarettes et la consommation d’aliments industrialisés.

L’hypertension artérielle reste partout une maladie assez fréquente. L’OMS par exemple a rapporté en 2015 qu’un homme sur quatre et une femme sur cinq avaient une HTA^45^. Une revue systématique ayant résumé les résultats de 242 études de 45 pays à revenu moyen et faible a trouvé une prévalence globale de 33.1%^46^. L’Afrique possède la prévalence la plus élevée d’HTA parmi toutes les régions de l’OMS pour les adultes de plus de 25 ans^47^. Une étude menée dans quatre pays d’Afrique subsaharienne a rapporté une prévalence de 25.9%^48^. Dans notre étude, nous constatons que la proportion des malades hypertendus est proche de la prévalence décrite par les autres auteurs. L’étude de Katchunga^22^ menée au Sud-Kivu, a noté une augmentation significative de la prévalence standardisée selon l’âge de l’HTA de 2012 à 2016 (19.0% vs 18.0% ; OR = 1.05 (1.02–1.08) ; *p* < 0.0001). Les résultats de notre étude paraissent légèrement supérieurs à ces derniers. Ceci pourrait s’expliquer par le fait que notre étude n’a interrogé que des patients se reconnaissant déjà malades. Notons par ailleurs que dans notre étude comme dans celle de Katchunga, la prévalence de l’HTA reste plus élevée en milieu urbain qu’en milieu rural.

Aussi, pour ce qui est de la proportion des cas d’HTA, nous pensons que cela serait liée aux différences climatiques de la région orientale de la RDC : région montagneuse où l’hypoxie et la polyglobulie secondaires à l’altitude impliquent certainement une augmentation de la viscosité sanguine et, donc, des résistances vasculaires qui à leur tour augmentent la pression artérielle^32^.

Le diabète est aussi une maladie émergente en Afrique dans un contexte de transition épidémiologique. Selon l’OMS, sa prévalence est de 8.5% en 2014^45,49^ tandis que le World Bank a trouvé une prévalence de 88.0% en 2019 chez les adultes de 20 ans et plus, dans le monde ; de 5.4% et 5.3% respectivement en Afrique Sub-saharienne et dans les pays à faible revenu. Elle était estimée à 6% en RD Congo^50^. Au Sud-Kivu, le diabète était plus retrouvé en milieu urbain que rural (4.9% vs 3.2% ; *p* < 0.001)^32^. En comparant ces prévalences avec les proportions de notre étude on trouve que, bien qu’elle soit plus élevée en milieu urbain qu’en milieu rural, la proportion du diabète chez nos enquêtés est élevée (7.9%).

### Choix thérapeutiques des hypertendus et diabétiques

Le recours aux FOSA intégrées est l’itinéraire thérapeutique initial le plus couramment utilisé par plus de la moitié des répondants dans les deux zones de santé. Cette observation a aussi été faite par d’autres auteurs dans des études similaires notamment dans la ville de Lubumbashi^51^.

L’automédication, un des itinéraires thérapeutiques émergeant des entretiens avec les parties prenantes, est un recours thérapeutique informel utilisé comme alternative. Ce résultat est similaire à celui rapporté à Lubumbashi en RDC où 54.6% des sujets recourent à l’automédication^52^. Ceci corrobore aussi les résultats de l’étude sur les déterminants des recours aux soins dans la ville de Cotonou au Benin où 58.5% faisaient recours à l’automédication^53^. D’autres études ont montré que l’automédication est plus pratiquée en milieu rural qu’en milieu urbain^51,52^.

L’importance de l’automédication mérite désormais une considération particulière, surtout que la présente étude révèle par ailleurs que plus de la moitié des personnes enquêtées la juge efficace. Elle s’impose comme une pratique à rationaliser plutôt que d’essayer de l’interdire en vain sous toutes ses formes^52^. Dans cet ordre d’idées, une option serait de focaliser les efforts de régulation sur une liste limitée de médicaments dont les contre-indications et les risques sont majeurs^54^ et de développer des programmes de promotion des pratiques rationnalisées de l’automédication pour des problèmes de santé moins sévères et fréquents chez les malades chroniques.

Des résultats de notre étude, il ressort que les personnes souffrant des pathologies chroniques comme le diabète et l’hypertension artérielle optent aussi pour d’autres itinéraires thérapeutiques comme les chambres de prière et la consultation d’un tradipraticien.

Cela serait lié à la précarité financière mise en évidence par plusieurs études dans les zones étudiées : les effets à long terme de l’insécurité, des guerres à répétition ont rendu difficile l’accès financier aux soins du fait de la paupérisation de la population dans l’Est de la RDC^20,28,33^.

D’autres études menées en Ouganda et en Guinée évoquent le même argument en s’appuyant sur le fait que le coût des services sanitaires formels est plus élevé que l’automédication ou la prise de produits traditionnels dans le cas du diabète de type 2^55,56^.

Le rôle que jouent les proches des patients diabétiques est notable du fait qu’ils contribuent à un accompagnement du patient concernant son traitement médical, les respects des mesures hygiéno-diététiques et parfois un soutien financier pour assurer le suivi médical (contrôles médicaux, achat de médicaments, hospitalisation)^57,58^.

Le choix porté sur la médecine traditionnelle et complémentaire par ces patients chroniques est encouragé voire entretenu par les membres de familles : dans plusieurs études, il ressort que certaines raisons motivent cette tendance retrouvée dans notre étude.

Premièrement, l’accessibilité financière est un critère incontournable : les patient peuvent discuter du prix de leur traitement avec les médecins traditionnels et dans certains cas les personnes indigentes peuvent être soignées gratuitement^55,59^.

Deuxièmement, la relation soignant-soigné serait plus forte entre les personnes souffrant de pathologies chroniques puisqu’elle tient compte du contexte socio-culturel et respecte les besoins des patients. Les tradipraticiens offrent ainsi une alternative thérapeutique adaptée au contexte^60^.

Néanmoins, certaines études nuancent en montrant que la relation soignant-soigné serait plus équilibrée quand le soignant est un(e) infirmier(e) : le temps d’écoute est plus long, la communication des informations plus détaillée au grand avantage des patients dont le traitement est parfois complexe et les pathologies sujettes à de fréquentes complications^61^.

Troisièmement, les stratégies utilisées par les médecines traditionnelles et complémentaires convainquent mieux les personnes en se basant sur des croyances (maladies dues aux esprits), des arguments selon lesquels les pathologies chroniques ne peuvent être prises en charge que par la médecine traditionnelle. Une revue systématique a trouvé que la spiritualité était liée à l’autogestion des patients atteints de maladies chroniques^62^.

L’une des formes particulières de médecine complémentaire retrouvées dans notre étude est celle des « chambres de prière », de petites églises (parfois même au domicile du pasteur) dans lesquelles les patients, ayant des pathologies chroniques entre autres, se font soigner par des prières. Selon les propos des acteurs enquêtés sur terrain, certains vont même jusqu’à « l’hospitalisation » des patients dans leurs chambres de prière.

Ces différents recours à la médecines traditionnelle ou complémentaire comme les chambres de prière pourraient enfin s’expliquer par la réponse inappropriée du système de soins de santé moderne aux attentes des patients diabétiques ou hypertendus : l’approche de soins actuellement développée aux différents échelons de ce système de soins semble plus adaptée à la prise en charge des problèmes de santé aigus. Surtout en première ligne, les soignants n’intègrent pas encore les aspects socioculturels et économiques des patients dans leur prise en charge et ce particulièrement pour les pathologies chroniques.

## Conclusion

Cette étude a montré que le diabète et l’HTA sont deux maladies chroniques relativement fréquentes dans les ZS de Walungu et Bagira à l’Est de la RDC. Pour leurs soins, hormis l’utilisation des formations sanitaires intégrées, les patients atteints de l’une et/ou l’autre de ces maladies recourent à l’automédication, aux chambres de prière, aux tradipraticiens, comme alternative. Il s’agit là des pratiques largement répandues aussi bien dans les zones de santé rurales qu’urbaines. Il convient de les rationaliser et de les formaliser dans le système de santé plutôt que de les ignorer comme c’est le cas actuellement. Cette rationalisation doit s’inscrire dans une vision plus globale de l’application de la politique nationale de la santé qui doit promouvoir l’intégration de ces différents acteurs, en améliorant la collaboration de ces derniers avec les personnels des structures formelles pour qu’ils prennent conscience de leurs limites dans la pratique médicale. Cette étude offre des arguments pour la formulation d’une proposition de réorganisation du système de santé dans la prise en charge des maladies chroniques.
